# Ge_1−x_Sn_x_ alloys: Consequences of band mixing effects for the evolution of the band gap Γ-character with Sn concentration

**DOI:** 10.1038/s41598-019-50349-z

**Published:** 2019-10-01

**Authors:** Timothy D. Eales, Igor P. Marko, Stefan Schulz, Edmond O’Halloran, Seyed Ghetmiri, Wei Du, Yiyin Zhou, Shui-Qing Yu, Joe Margetis, John Tolle, Eoin P. O’Reilly, Stephen J. Sweeney

**Affiliations:** 1 Advanced Technology Institute and Department of Physics, University of Surrey, Guildford, GU2 7XH United Kingdom; 2 Tyndall National Institute, Lee Maltings, Cork, T12 R5CP Ireland; 3 School of Chemistry, University College Cork, Cork, T12 YN60 Ireland; 4 Department of Electrical Engineering, University of Arkansas, Fayetteville, AR 72701 USA; 5 Department of Electrical Engineering, Wilkes University, Wilkes-Barre, PA 18766 USA; 6 ASM, 3440 East University Drive, Phoenix, Arizona 85034 USA; 7 Department of Physics, University College Cork, Cork, T12 YN60 Ireland

**Keywords:** Electronic structure, Silicon photonics

## Abstract

In this work we study the nature of the band gap in GeSn alloys for use in silicon-based lasers. Special attention is paid to Sn-induced band mixing effects. We demonstrate from both experiment and ab-initio theory that the (direct) Γ-character of the GeSn band gap changes continuously with alloy composition and has significant Γ-character even at low (6%) Sn concentrations. The evolution of the Γ-character is due to Sn-induced conduction band mixing effects, in contrast to the sharp indirect-to-direct band gap transition obtained in conventional alloys such as Al_1−x_Ga_x_As. Understanding the band mixing effects is critical not only from a fundamental and basic properties viewpoint but also for designing photonic devices with enhanced capabilities utilizing GeSn and related material systems.

## Introduction

In the field of photonics, it is a long-held goal to realise all-silicon based technologies. While silicon (Si) has been instrumental to the development of electronics, its use in photonics has been limited to passive components such as waveguides and devices such as photodetectors and modulators. The fundamental limitation of Si is its indirect band gap which significantly decreases the probability of light emission compared to direct band gap semiconductors such as gallium arsenide (GaAs)^1^. Various strategies have been developed to overcome this fundamental limitation, such as direct epitaxial growth and heterogeneous integration of conventional III-V semiconductors on Si^2–6^. Germanium-tin (GeSn) offers the potential to engineer the first tunable group IV semiconductor alloy with a fundamentally *direct* band gap^7,8^. In recent years this material system has received considerable attention as a viable pathway for the next generation of Si-compatible optoelectronic devices. Most significantly GeSn offers the potential to realise an efficient infrared light source on Si^9–11^, with applications in optical interconnects and lab-on-chip trace gas detection. In addition narrow band gap semiconductors on Si offer opportunities for tunnelling enhancement in devices such as tunnelling-field effect transistors (TFETs)^12,13^ for which GeSn is considered as a potential candidate system^14,15^. Also, Si has demonstrated enhanced nonlinear effects which is important for nonlinear THz applications^16^, and these nonlinear effects are expected to get stronger in narrow band gap materials such as GeSn and its alloys^17^. Due to recent advances in the growth of high crystalline quality GeSn and the diverse nature of its applications, the focus on GeSn alloys has gathered enormous pace^18–23^. Critical to the development of these materials is obtaining a detailed understanding of the band structure evolution of GeSn and the influence of Sn concentration on its electronic and optical properties.

**Figure 1:**
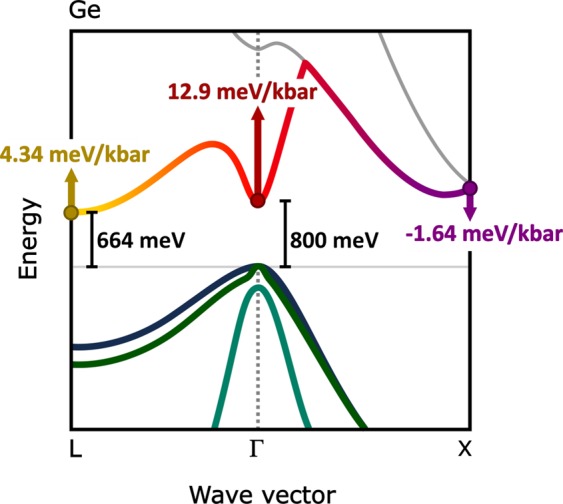
Schematic illustration of the pure bulk Ge bandstructure at 300 K with the Γ, X and L pressure coefficients indicated with respect to the valence band edge at the Γ-point.

Germanium (Ge) is fundamentally an indirect band gap semiconductor. The indirect energy gap, E_g_(L) = 664 meV at 300 K, is between the highest valence band (VB) state at Γ and the lowest conduction band (CB) states at L. The lowest CB Γ state lies 136 meV above the L CB minimum^24^. The bandstructure of bulk Ge is illustrated in Fig. 1. The present discussion in the literature regarding the band gap evolution of GeSn alloys is based on the assumption that with increasing Sn composition there is a sharp transition from GeSn being an indirect band gap material to a direct one^18,19,25–27^. However, there is a large degree of uncertainty for the Sn concentration at which this transition occurs, with typical values ranging from ~6–11% Sn^28–31^. Moreover, this analysis neglects alloy induced band mixing effects, which could render the assumption of a sharp direct to indirect band gap invalid. For an alloy with strong band mixing effects, rather than labelling a semiconductor as “direct” or “indirect” it is more appropriate to consider the *fractional* Γ-character of the CB edge (CBE). Thus, the fractional Γ-character describes what proportion of the lowest conduction states have Γ-like (direct) character. Both the optical gain in a laser^32^ and the tunnelling rate in a TFET at the band edge^33^ depend directly on the square of the Kane momentum matrix element, |P|^2^, which describes the coupling between the lowest conduction and highest valence states. This, in turn, is directly proportional to the fractional Γ character of the lowest CB states^34^. Therefore, understanding the conduction Γ-L state mixing in GeSn is of central importance from a fundamental physics perspective, but also for designing the next generation of group IV electronic and optoelectronic devices.

In this work, we show from both experiment and ab-initio theory that there is *not* a sharp direct to indirect transition in GeSn. Instead, the evolution of the GeSn optical and electronic properties is determined by band mixing effects between the Γ and L CB states. It is therefore more appropriate to discuss the nature of the GeSn band gap in terms of the fractional Γ character of the alloy fundamental band gap E_g_ rather than in the widely accepted frame of a sharp indirect to direct band gap transition. In earlier theoretical studies these features have been neglected either through the choice of supercell^35^, or through insensitivity of the model to these effects (e.g. treating the material in the virtual crystal approximation)^36^. Previous experimental works such as those based on photoreflectance are also insensitive to indirect transitions^37–39^.

Using high hydrostatic pressure measurements, we address the question of the fractional Γ character of the CBE in GeSn alloys. The direct and indirect band gaps in Ge have distinctly different pressure dependencies, as illustrated in Fig. 1. The value of the pressure coefficient may therefore be used to distinguish between direct and indirect band gap transitions in GeSn alloys. GeSn p-i-n diode samples with Sn concentrations of 6.1%, 6.4% and 9.2% (±0.3%) are investigated using pressure dependent photovoltage spectroscopy measurements of the absorption edge. As discussed in detail below, with only 6% Sn, the pressure coefficient of the GeSn absorption edge, at 9.2 meV/kbar, is intermediate between that of the indirect L gap (4.3 meV/kbar) and that of the direct Γ gap (12.9 meV/kbar), providing clear evidence of the substantial Γ character of E_g_ below what is typically predicted as the indirect-direct band gap crossover in GeSn alloys^28–31^. Furthermore, we show that with increasing Sn concentration, the pressure coefficient increases monotonically, approaching that of the direct Γ gap. The intermediate value pressure coefficient for 6% Sn is analogous to that observed previously in dilute-nitride GaInN_x_As_1−x_ systems, where the reduced pressure coefficient was used to identify a band anti-crossing interaction between the GaInAs host matrix Γ CBE and N-related localised states lying above the CBE^34,40^.

These experimental measurements are accompanied by hybrid functional density functional theory studies on structures that allow for Γ- and L-state mixing effects and account for alloy induced features on a microscopic level. The significant Γ character and transitional behaviour in the pressure coefficient observed in the experiment indicates band mixing effects between the CB states. The experimental findings are supported by our atomistic, first-principles calculations. The pressure dependent band edge calculations reveal very similar trends when compared to the experimental results, supporting the Γ-L state mixing effect. Moreover, our theoretical calculations give insight into the origin of the mixing between the different CB states.

Overall, our combined experimental and theoretical work provides evidence of substantial Γ (direct-gap) character in GeSn, even at compositions where the band gap is expected to be indirect. The dominant Γ character, present even with 6% Sn concentrations is indicative of CB mixing effects. The analysis and conclusions presented here are fundamentally different to the usual assumption of a sharp indirect-to-direct band gap transition in GeSn alloys. Such band mixing can therefore lead to improved optical properties at lower Sn concentrations than would otherwise be expected, as well as opening an efficient tunnelling path in TFETs. But, at the same time the intrinsic alloy fluctuations may lead to an inhomogeneous broadening of the band edges, which impacts the TFET turn-on rate, reduces electron mobility and broadens the gain spectrum thereby reducing the peak gain. Therefore, a higher carrier density for transparency and threshold may be required in lasers. Although the composition dependence of mixing effects on broadening is uncertain, mixing is likely to be strongest near the indirect/direct band gap “crossover” and to become less important with increasing Sn content.

## Results and Discussion

The optical properties of GeSn are investigated using GeSn p-i-n photodiodes grown with low-temperature Chemical Vapour Deposition (CVD). The Double Heterostructure (DHS) p-i-n photodiodes were grown on a Si substrate using a thick strain relaxed p-doped Ge buffer as a virtual substrate. This was followed by the deposition of a 200 nm unintentionally doped GeSn film and an n-doped Ge cap layer. The Sn concentrations in the GeSn layer were measured as 6.1%, 6.4% and 9.2% using X-ray diffraction, with residual compressive strains of −0.5%, −0.4% and −0.5%. The high crystal quality of the GeSn layer was confirmed using cross-sectional transmission electron microscopy. Further details of the growth can be found elsewhere^41^. The Sn concentrations correspond to the range of values where the contemporary literature predicts the indirect to direct band gap crossover to occur in GeSn^28–31^. As we explain below, due to band mixing effects it is more appropriate to discuss the band gap character rather than the indirect or direct nature of the band gap over a critical composition range. An important metric is the fractional Γ character, which describes here the proportion of the CBE state that projects onto Γ-like bulk Ge states. The band edge optical recombination rate, tunnelling rate and optical gain are all expected to increase monotonically with increasing Γ character^32–34^.

The Γ-character is determined experimentally using high hydrostatic pressure. Subjecting a crystalline solid to hydrostatic pressure reversibly compresses the sample while preserving crystal symmetries. The reduced lattice spacing modifies important features of the band structure such as the relative positions of the CB minima. Referenced from the top of the VB, the pressure coefficients of the Γ and L conduction band minima have been predicted theoretically as 12.9 meV/kbar and 4.34 meV/kbar respectively in Ge^42^ (Fig. 1), consistent with experimental values^43–45^. The pressure coefficient of the relevant Γ states in α-Sn is comparable, (15.7 meV/kbar), and so the Γ pressure coefficient of the measured GeSn samples are not expected to differ significantly from that of pure Ge^42^. The large difference in the pressure coefficients between Γ and L allows the Γ-character of the effective band edge to be determined. Hydrostatic pressure is therefore uniquely suited to investigate the fundamental properties of emerging and complex material systems such as GeSn^40^.

**Figure 2:**
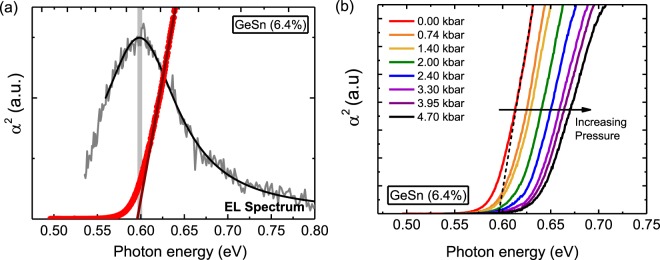
(**a**) Electroluminescence and the square of the absorption coefficient measured at atmospheric pressure for the Ge_0.936_Sn_0.064_ photodiode. (**b**) Absorption spectra (α^2^) under hydrostatic pressure, measured at room temperature.

The band edge character of GeSn is determined here by measuring the pressure coefficient of the effective band edge. For the purpose of this work, the effective band edge refers to the aggregate effect of the low energy CB states, which determines the optical properties of GeSn. The pressure coefficient was determined by extracting the absorption spectrum from photovoltage measurements and measuring the energy shift of the band edge under high pressure. Photovoltage spectra were obtained using a lock-in-amplifier and a Bentham grating-based monochromator with an integrated light source to select wavelengths. The monochromated light was modulated using an optical chopper. The linewidth for each wavelength measurement was 10 nm. For the high-pressure measurements, the GeSn photodiodes were housed inside a CuBe pressure cell with light focused through a sapphire window onto the device. Hydrostatic pressures of up to 5 kbar were applied using a compression system with helium gas as the pressure medium. Due to alloy disorder, the absorption spectrum will be composed of a complex distribution of direct and indirect-like transitions across a wide wavelength range. It is therefore essential to have a strong band edge feature in the resultant absorption spectrum which can be consistently measured under pressure. Taking the square of the absorption spectrum, plotted as a function of photon energy, and extrapolating the falling slope to the energy axis (Tauc’s equation^46^) gives good agreement with the peak of the electroluminescence spectrum measured at ambient pressure (Fig. 2(a)).

Due to the small aperture window in the high pressure cell, it was not possible to obtain electroluminescence under pressure owing to the low signal-to-noise ratio; however, this extrapolation procedure provides a strong and consistent method to establish the pressure coefficient of the effective band edge. Extrapolation of α^2^ has been used previously in the literature to determine the compositional dependence of the direct band gap of GeSn^47,48^. However, as revealed by our high pressure measurements detailed below, the compositional dependence of the absorption edge is somewhat more complex, and we find from the pressure coefficients that what is measured as direct gap absorption can actually be absorption from states with mixed Γ (direct) and L (indirect) character.

**Figure 3:**
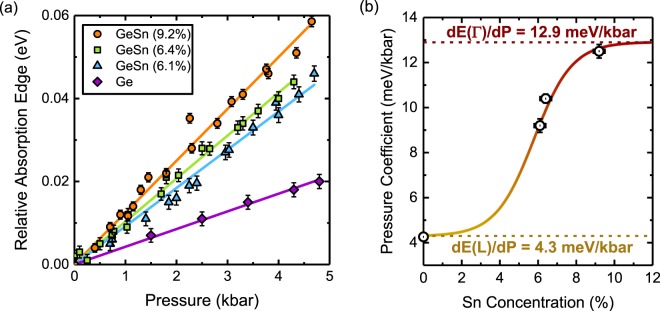
(**a**) The energy shift of α^2^ as a function of pressure from atmospheric pressure for Ge and GeSn photodiodes. The gradients of the pressure dependence give the pressure coefficients of the band edge, which are plotted as a function of Sn concentration in (**b**). The sigmoid plot is a guide to the eye.

The method for determining the pressure coefficient of the effective band edge is illustrated in Fig. 2(b). With increasing pressure, the absorption edge shifts to higher energies, due to the positive pressure coefficients associated with the Γ- and L- CB minima. At each pressure, we observe a strong linear region in the α^2^ data. The measured relative shifts of the band edge as a function of pressure for the three GeSn photodiodes are presented in Fig. 3(a). In each case, there is a strong linear relationship between the shift of the absorption edge and the applied pressure value. A pressure coefficient can therefore be reliably determined for each sample. For comparison, the movement of the absorption edge of a commercial Ge photodiode is presented. As can be seen from Fig. 3(a), the pressure coefficient of 4.3 meV/kbar, derived for the Ge photodiode, is in good agreement with the pressure coefficient of the L conduction minima in pure Ge. The derived pressure coefficients of the GeSn photodiodes are 9.2 meV/kbar, 10.4 meV/kbar and 12.5 meV/kbar for the 6.1%, 6.4% and 9.2% Sn samples, respectively.

There are a number of notable features in these results. Firstly, the pressure coefficient of 9.2 meV/kbar for the 6.1% Sn sample is just over midway between the L-point value (4.3 meV/kbar) and the Γ direct gap value (12.9 meV/kbar) of pure Ge. This provides evidence for substantial Γ character at the band edge in GeSn, even at a composition where the band gap is generally predicted to be indirect. The dominant Γ character of the band edge, even with such a low Sn concentration is, as we show below, evidence of CB mixing effects. Secondly, with increasing Sn concentration, there is a monotonic increase in the pressure coefficient of the band edge. Significantly, there is no sharp transition in the pressure coefficient with Sn concentration, as would be expected in conventional alloys such as GaAs^49^, AlGaAs^50^, InGaSb^51^ and AlInAs^52^. Instead, we observe a continuous increase in the Γ character with Sn concentration. This evolution is illustrated in Fig. 3(b), which plots the pressure coefficient as a function of Sn concentration, illustrating the asymptotical approach of the GeSn pressure coefficient to that of the Γ CB minimum of Ge.

### Theoretical framework

The experimental pressure results presented in the previous section provide a clear indication of Γ-L mixing in GeSn alloys with increasing Sn content. This challenges the widely accepted view of a sharp indirect-to-direct band gap transition^29,35,53,54^. Here, we use density functional theory (DFT) calculations in the Heyd Scusceria Ernzerhof (HSE) hybrid functional scheme^55–57^ to further support, from a first principles approach, the experimental findings of a continuous evolution of the band gap from being indirect to direct. The details of our HSE-DFT calculations, implemented in the Vienna ab-initio software package (VASP)^58,59^, are given in the “Methods” section below. However, before turning to the results of our calculations, it is useful to discuss briefly theoretical approaches to analyse the evolution of the band structure and potential (conduction) band mixing effects in an alloy such as GeSn. A widely used approach to address this question is band unfolding, where the bands of the supercell are folded back to the larger original primitive first Brillouin zone^60–62^. From the resulting unfolded band structure, the **k** character of the energetically lowest lying CB state in the Brillouin zone (e.g. at Γ- and/or the L-point) can be identified. Analysing the spectral weights of this state at different **k**-points can then give insight into band mixing effects. However, depending on how many states are involved in the band mixing, it can be difficult to identify these contributions easily and clearly. For example, because there are 4 L states in the unfolded band structure, and the lowest state at Γ can mix with all 4 of them, only a small fraction (25%) of this L character is associated with the one L point typically shown in an unfolded band structure. A quick visual inspection of the unfolded band structure can then significantly underestimate the amount of mixing present. Here we employ a different approach to study the evolution of the CBE character in GeSn alloys, namely performing hydrostatic pressure dependent DFT calculations. In doing so we obtain the same information as from the band unfolding, but with the additional benefits that we can (i) identify band mixing effects more clearly, given the large differences in Γ, L and X pressure coefficients and (ii) directly compare our theoretical results to the experimental data.

### Band structure calculations: Pressure dependence of band gap

**Figure 4:**
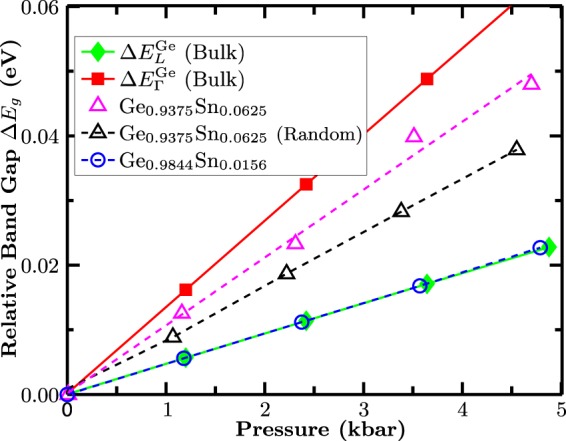
The relative band gap Δ*E*_*g*_ as a function of the applied pressure from hybrid-functional density functional theory calculation. Calculations have been performed for GeSn supercells with different Sn contents. As a reference, calculated data for pure Ge bulk systems are also given.

To analyze the pressure dependence of the band gap in free-standing GeSn alloys and to gain insight into the fundamental electronic structure properties of these systems from theory, we proceed in the following way. First, and key to this study, 16- and 64-atom supercells (SCs) have been chosen, for both of which the bulk Ge CB L and X states fold to the SC Γ point. With these states folding back in this way, band mixing can then occur between Γ and the high symmetry L and X CB states. This is in contrast to previous DFT calculations where SCs have been used to exactly avoid this mixing^35^ or where mixing effects have not been considered in the analysis of the band structure^36^. Moreover, and in line with the experimental considerations, it is important to understand how the electronic structure and therefore the Γ–L conduction state mixing evolves with Sn content. Taking these two factors into account, Sn composition and Γ−L-CB state mixing, the following cells have been investigated in our theoretical analysis. To study very low Sn contents a 64 atom SC has been constructed where one Ge atom is replaced by a Sn atom (Ge_63_Sn_1_). This leaves us with a system of only 1.56% Sn (Ge_0.9844_Sn_0.0156_). Given that the experimental data, Fig. 3(a), shows clear indication of Γ−L state mixing for GeSn alloys with 6% Sn, we have primarily targeted this composition in the calculations. To analyze the importance of the alloy microstructure on the results for this composition, two different cells with nominally the same Sn content (6.25% Sn) have been generated. The first SC is again a 64 atom SC. Here, 4 Ge atoms have been replaced at random by 4 Sn atoms (Ge_60_Sn_4_). In addition, we have considered a 16 atom SC (face centred cubic (fcc) cell) with one Sn atom (Ge_15_Sn_1_). In this case, due to the small SC size and the periodic boundary conditions, a very ordered arrangement of the Sn atoms has been constructed. Below we discuss in more detail the impact of the SC size on the results. Using these cells, we have studied the composition and pressure dependence of the band gap energy E_g_ of the different systems. To do so, the structures have been initially relaxed (cell shape, volume and internal degrees of freedom of the atoms) to find the equilibrium lattice constant. To mimic the effect of pressure on the cell, the equilibrium lattice constant has been reduced and the internal atomic positions have been relaxed again at each pressure. Figure 4 shows the results of these calculations.

Following the experimental approach, we have calculated here the *relative* band gap change ΔE_g_ with pressure, meaning that the band gap shift with respect to the zero pressure data (equilibrium structure) is displayed. As a reference, we have performed the same calculation for a two atom, pure Ge cell. Again, this procedure is similar to the experimental setup. From the pure Ge cell the pressure dependence of the Ge band gap at the Γ-point, $\Delta {E}_{\Gamma }^{{\rm{Ge}}}$ (Bulk) (red filled squares), and the fundamental band gap, $\Delta {E}_{{\rm{L}}}^{{\rm{Ge}}}$ (Bulk) (green filled diamonds), between the VB edge (${\Gamma }_{8{\rm{v}}}$-state) and the CBE at the L-point (L_6c_-state), have been extracted. The pressure coefficient (dE(Γ)/dP)^theo^ for $\Delta {E}_{\Gamma }^{{\rm{Ge}}}$ (Bulk) is (dE(Γ)/dP)^theo^ = 13.3 meV/kbar. For the indirect band gap $\Delta {E}_{{\rm{L}}}^{{\rm{Ge}}}$ (Bulk) a pressure coefficient of (dE(L)/dP)^theo^ = 4.66 meV/kbar is calculated. These numbers are in very good agreement with the values ((dE(Γ)/dP)^exp^ = 12.9 meV/kbar, (dE(L)/dP)^exp^ = 4.34 meV/kbar) given above. Starting with the 1.56% Sn data (Ge_0.9844_Sn_0.0156_, blue open circles) we find that the pressure dependence of the band gap energy at this very low Sn content (4.75 meV/kbar) is extremely close to the pressure coefficient of the indirect band gap of pure Ge (green filled diamonds in Fig. 4), confirming an indirect band gap at very low Sn content. However, when turning to the 6.25% Sn systems (magenta and black open triangles in Fig. 4), the calculated band gap pressure dependence of the GeSn alloy shows very similar results when compared to the experimental data, cf. Fig. 3(a). A closer inspection of the 6.25% Sn system reveals that even though the 16 (magenta open triangles in Fig. 4) and the 64 (black open triangles) atom SCs have nominally the same Sn content, they exhibit clearly different pressure coefficients of 10.5 and 8.2 meV/kbar, respectively. This highlights that the alloy microstructure and the SC size noticeably affect the electronic structure of this system. This effect will be investigated and discussed further below, when band mixing effects are investigated in more detail.

Overall, the theoretical and experimental data show that by 6% Sn the alloy CBE has strong Γ character (>50% Γ in experiment and the 16-atom SC; >40% in the 64-atom SC considered). Consequently, when accounting for the CB Γ-L-state mixing in the calculations, our theoretical results are consistent with the experimental observation of a continuous transition from an indirect-to-direct band gap material. This aspect has been widely neglected in previous literature studies and sheds new light on the evolution of band gap in GeSn alloys with Sn content, clearly modifying the previous perception of a sharp indirect to direct band gap transition^29,35,53,54^.

### Band structure calculations: Band mixing effects

**Figure 5:**
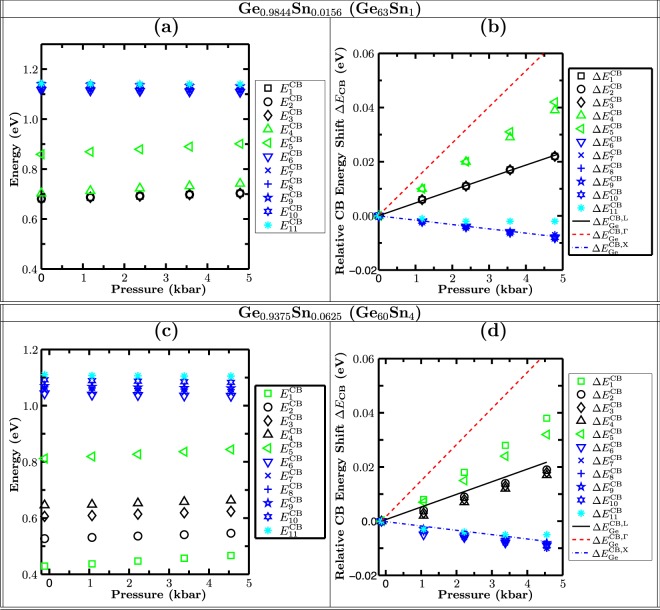
(**a**) Pressure dependence of the 11 energetically lowest zone centre conduction band (CB) states in Ge_0.9844_Sn_0.0156_ (64 atom supercell with 1 Sn and 63 Ge atoms, Ge_63_Sn_1_) with respect to the valence band (VB) edge energy. (**b)** Change in energies in (**a**) with pressure. Additionally, the pressure dependence of the pure Ge Γ- (red dashed), L- (black solid) and X- (blue dashed-dotted) gaps are also shown. **(c)** Same as (**a**) but for Ge_0.9375_Sn_0.0625_ (64 atom supercell with 4 Sn and 60 Ge atoms; Sn atoms randomly distributed; Ge_60_Sn_4_). (**d)** Same as in (**b**) for the Ge_0.9375_Sn_0.0625_ 64 atom supercell.

To further support this finding, we have studied the pressure dependence with respect to the VB edge of the first 11 CB states of the two 64 atom SCs considered here. The results of this study are displayed in Fig. 5. The first row, Fig. 5(a,b), shows data for the 1.56% Sn system (Ge_63_Sn_1_). In Fig. 5(a) the energy separation of the first 11 CB states with respect to the VB edge energy at each pressure is displayed. Figure 5(b) depicts the relative energy shift of the CB states with applied pressure. The results of the same analysis, but this time for the 6.25% Sn system, carried out with a random distribution of 4 Sn atoms in a 64 atom SC (Ge_60_Sn_4_), are depicted in Fig. 5(c,d).

### Band structure calculations: Band mixing effects at low Sn contents

We start with the analysis of the 1.56% Sn system (Fig. 5(a,b)). When looking at the relative energetic positions of the different CB states with respect to the VB edge at each pressure, Fig. 5(a), we find that the first three CB states ($\Delta {E}_{1}^{{\rm{CB}}}$, $\Delta {E}_{2}^{{\rm{CB}}}$, $\Delta {E}_{3}^{{\rm{CB}}}$) are degenerate in energy. When looking at their pressure dependence, Fig. 5(b), these states exhibit the pressure dependence of the CB L-state in pure Ge (black solid line in Fig. 5(b)), consistent with the band gap data in Fig. 4 for 1.56% Sn. Before turning to Δ${E}_{4}^{{\rm{CB}}}$ and Δ${E}_{5}^{{\rm{CB}}}$ (triangles in Fig. 5(a)), we first look at Δ${E}_{6}^{{\rm{CB}}}$ to Δ${E}_{11}^{{\rm{CB}}}$. Again, these states are almost degenerate in energy. Furthermore, they are energetically clearly separated from Δ${E}_{1}^{{\rm{CB}}}$ to Δ${E}_{5}^{{\rm{CB}}}$. We attribute Δ${E}_{6}^{{\rm{CB}}}$ to Δ${E}_{11}^{{\rm{CB}}}$ to X–like states. This is confirmed by their pressure dependence, cf. Fig. 5(b), which follows closely the pressure dependence of the Ge bulk X-CB state (blue dashed dotted line). A closer inspection of the pressure dependence of Δ${E}_{11}^{{\rm{CB}}}$ (light blue stars) reveals a slightly larger pressure coefficient than the other X-like states $\Delta {E}_{6}^{{\rm{CB}}}$ to $\Delta {E}_{10}^{{\rm{CB}}}$. Before looking at this behaviour in detail, we examine the pressure dependence of Δ${E}_{4}^{{\rm{CB}}}$ and Δ${E}_{5}^{{\rm{CB}}}$ first. Here, these two states exhibit pressure coefficients which are intermediate between the pressure coefficients of Ge bulk CB L- (black solid) and Γ-states (red dashed). In the SCs considered here, one expects in general 4 L-like states, with three having *p*-like symmetry about the Sn atom and one having *s*-like symmetry. Therefore Δ${E}_{4}^{{\rm{CB}}}$ and Δ${E}_{5}^{{\rm{CB}}}$ are a mixture of Γ- and L-states with *s*-like symmetry on the Sn site. Additionally, given that Δ${E}_{11}^{{\rm{CB}}}$ shows a slight deviation from the pure Ge X-like bulk pressure dependence we expect also a contribution from this X-like state to Δ${E}_{4}^{{\rm{CB}}}$ and Δ${E}_{5}^{{\rm{CB}}}$. The predicted mixing of the different states is further supported by the observation that the sum of the pressure coefficients of Δ${E}_{4}^{{\rm{CB}}}$, Δ${E}_{5}^{{\rm{CB}}}$ and Δ${E}_{11}^{{\rm{CB}}}$ is within 1 meV/kbar of the sum of the pressure coefficients of the bulk Ge Γ-, L- and X-states. Consequently, our data clearly indicate band mixing effects between *s*–like L-, Γ- and X-CB states. However, for the Sn content of 1.56% the mixed states lie above the CB minimum, and the CBE is still made up of states that are L-like in character. To understand the effect of increasing Sn content on band mixing effects and, thus, the connected consequences for the band gap of GeSn alloys, we turn now to the 64 atom SC with a random distribution of 4 Sn atoms.

### Band structure calculations: Band mixing effects at higher Sn contents

In comparison to the 1.56% Sn case, cf. Fig. 5(b), the striking difference in the 6.25% Sn case is that the lowest CB state (Δ${E}_{1}^{{\rm{CB}}}$, green squares), Fig. 5(c,d), has a pressure coefficient intermediate between the Ge bulk CB Γ- (red dashed line) and L-state (black solid line), with a second state, Δ${E}_{5}^{{\rm{CB}}}$ (green triangles) also having an intermediate pressure coefficient. Again, this indicates Γ-L-state mixing due to Sn incorporation in the SC. Looking now at the other CB states, Fig. 5(c), Δ${E}_{2}^{{\rm{CB}}}$ to Δ${E}_{4}^{{\rm{CB}}}$ are all L state related, as confirmed by their pressure dependence, Fig. 5(d). Due to alloy disorder, their degeneracy is lifted. $\Delta {E}_{6}^{{\rm{CB}}}$ to Δ${E}_{11}^{{\rm{CB}}}$ are again X-like in character, with some mixing from Γ- and L-like states, as can be seen from their pressure dependence, Fig. 5(d). Our results on the 16 atom fcc SC with just 1 Sn atom, but nominally the same Sn content, give similar trends in the pressure dependence of the band gap as the 64-atom random alloy SC (cf. Fig. 4). But, as one can infer from Fig. 4, a higher pressure coefficient of the band gap is observed in the 16 atom fcc SC. We attribute this to the small SC size and the resulting long-range correlations (ordering) introduced by the periodic boundary conditions, which leads to increased Γ character for the lowest conduction state in the 16 atom fcc SC compared to the 64-atom random structure considered. This comparison highlights that further studies are required to understand the impact of the SC size on the CB mixing effects in full detail. To do so, systems with several hundred to thousands of atoms would need to be studied, in order to minimize the influence of long range ordering arising from the periodic boundary conditions of the simulation cell. However, systems of such size are beyond the capabilities of standard DFT approaches. Consequently, empirical models, such as the tight-binding or (30 band) **k**.**p** models are required. However, these empirical methods have to be benchmarked against, for example, DFT results to obtain reliable results. Therefore, our presented HSE-DFT calculations not only provide new insight into the electronic structure of GeSn alloys, they can now also serve as a starting point for developing empirical models to study the electronic and optical properties of these systems. Overall, even without using these very large SCs, our calculations support that band mixing effects in GeSn alloys lead to a *continuous evolution* of the material from being an indirect band gap system to a direct one.

To summarise, in this work we investigated the compositional dependence of the band gap character of GeSn both from theory and experiment. On the experimental side, three GeSn alloys were studied with Sn concentrations between 6–10%. The experimental approach uses hydrostatic pressure to reversibly modify the electronic band structure of GeSn. The substantial difference in the CB pressure coefficients of Γ and L allow their contributions to the effective band edge to be determined under pressure. Measuring the effect of hydrostatic pressures on the absorption edge, we derived pressure coefficients of the effective band edge, as a function of Sn composition. In these results we observe a continuous evolution in the band gap character, with increasing Γ-like character as a function of Sn concentration. Considerable Γ-like character is observed at 6% Sn in the GeSn alloy, contrary to the general expectation that this alloy should have an indirect band gap. The dominant Γ-like character and its continuous compositional dependence are indicative of band mixing effects. To investigate the impact of band mixing on the electronic properties of GeSn further, the band structure was calculated using hybrid functional DFT. These calculations further supported the experimentally observed band mixing effects and thus a continuous evolution of the band gap from being indirect to direct. Therefore, our results offer a detailed fundamental understanding of the electronic and optical properties of GeSn alloys. Band mixing effects have important implications for the viability of future electronic and photonic devices based on GeSn alloys. One consequence of band mixing is improved optical properties at lower Sn concentrations than would otherwise be expected. However, mixing and random alloy fluctuations may lead to an intrinsic inhomogeneous broadening of band edges. This would broaden the gain spectrum, requiring a higher carrier density for transparency and threshold. Interband tunnelling could likewise be enhanced at lower Sn concentrations but with inhomogeneous broadening reducing the rate at which current increases with voltage. In addition, strong mixing effects will increase electron scattering in conventional electronic devices, thereby reducing electron mobility compared to that expected using a virtual crystal approximation. While the composition dependence of mixing effects is not yet known it is likely to be strongest near the indirect/direct band gap “crossover” and become less important with increasing Sn content.

## Methods

### Sample fabrication

The samples were fabricated into circular mesa structures with diameters of 100, 250 and 500 μm using photo lithography and wet etching processes. The wet chemical etch (HCl: H_2_O_2_: H_2_O = 1:1:20 at room temperature) showed a stable etching rate of 100 nm/min regardless of Sn composition. A 100-nm-thick SiO_2_ passivation layer was then deposited by plasma-enhanced chemical vapour deposition followed by the openings made for the metal contacts. The p- and n-type metal contacts consist of 10 nm Cr and 200 nm Au defined by metal deposition and liftoff processes.

### Photovoltage measurements

For the photovoltage measurements, light from a broadband source was selected using a Bentham TMc300 Triple Grating Monochromator. The photocurrent was measured using a Stanford Research Systems SR830 lock-in amplifier. The devices were mounted onto Transistor Outline (TO) headers. Thermoconductive epoxy was used as an adhesive with gold wire bonding to the n- and p- contacts. The device was loaded into a non-magnetic CuBe high pressure cell. Hydrostatic pressure was applied with a gas compression system (UniPress) using helium gas as an inert hydrostatic pressure medium. Optical access was provided through a small sapphire window in the high pressure cell and the light was focused onto the mesa. The absorption spectrum was derived from the photovoltage, after correcting for the output spectrum of the light source and system response. The absorption in the thin 50 nm Ge cap layer was assumed to be negligible and therefore neglected.

### Theory

All our density functional theory (DFT) studies have been performed within the Heyd Scuseria Ernzerhof (HSE) hybrid functional scheme, using the projector augmented-wave (PAW) method^63,64^. More specifically we apply here the HSEsol scheme^57^. The calculations have been carried out in the framework of the plane-wave-based ab initio package VASP^59,64^. For an accurate description of the CB and VB structures of both Ge and α-Sn we have used the following settings: the screening parameter μ was set to 0.2 Å^−1^ while the exact exchange mixing parameter is 0.3. To avoid artefacts from Pullay stresses when performing structural relaxations and connected electronic structure calculations, a large plane wave cut-off energy of 400 eV was used. For the bulk calculations, the underlying Γ-centered **k**-point grid is 6 × 6 × 6. For the SC calculations (16 atom face centred cubic cell, and 64 atom cubic cells) a 3 × 3 × 3 Γ-centered **k**-point mesh was applied. Our calculations include spin-orbit coupling (SOC) to achieve an accurate description of the α-Sn band structure at the Γ-point. The semi-core *d*-states in Ge and Sn were treated as core electrons, since several studies have reported that unfreezing them has a negligible effect on the electronic structure^35,65^. Within this theoretical framework the CB splitting between Γ-L states in Ge is 143 meV, which is in very good agreement with the literature data. Furthermore, for α-Sn we calculate a SOC energy ΔE_so_ of ΔE_so_ = 0.751 eV and a negative band gap of E_g_^Γ^ = −0.373 eV. Again these numbers are in good agreement with the experimental (ΔE_so_ = 0.8 eV^66^ E_g_^Γ^ = −0.413 eV^66^) and theoretical literature (ΔE_so_ = 0.681 eV^65^ E_g_^Γ^ = −0.519 eV^65^) data. We conclude that the DFT approach used is ideally suited to investigate GeSn alloys on a first-principles level and can form the basis for developing empirical (atomistic) models.
